# Bayesian inferences about the self (and others): A review

**DOI:** 10.1016/j.concog.2014.01.009

**Published:** 2014-04

**Authors:** Michael Moutoussis, Pasco Fearon, Wael El-Deredy, Raymond J. Dolan, Karl J. Friston

**Affiliations:** a Wellcome Trust Centre for Neuroimaging, UCL, United Kingdom; b Department of Psychology, University College London, United Kingdom; c School of Psychological Sciences, University of Manchester, United Kingdom

**Keywords:** Self-representation, Other-representation, Free energy minimisation, Active inference, Personality disorder, Paranoia

## Abstract

Viewing the brain as an organ of approximate Bayesian inference can help us understand how it represents the self. We suggest that inferred representations of the self have a normative function: to predict and optimise the likely outcomes of social interactions. Technically, we cast this predict-and-optimise as maximising the chance of favourable outcomes through active inference. Here the utility of outcomes can be conceptualised as prior beliefs about final states. Actions based on interpersonal representations can therefore be understood as minimising surprise – under the prior belief that one will end up in states with high utility. Interpersonal representations thus serve to render interactions more predictable, while the affective valence of interpersonal inference renders self-perception evaluative. Distortions of self-representation contribute to major psychiatric disorders such as depression, personality disorder and paranoia. The approach we review may therefore operationalise the study of interpersonal representations in pathological states.

## Highlights

• People may use Bayesian inference to update their own self-representation.
• Self- and other-representations may help predict outcomes of social interactions.
• The value of an outcome is essentially the prior belief that it can be achieved.
• ‘Active inference’ uses free-energy-minimization to achieve desirable outcomes.
• A positive self-representation may be a desirable outcome of active inference.

## 1 Introduction: Agency and the interpersonal self

The sense of self may be experienced at many levels – from the elementary, pre-verbal ‘minimal self’ that accompanies all conscious perception, to the purposeful, historically constructed ‘narrative’ self who takes action under the conscious guidance of goal and context. Research based on the idea of the brain as a probabilistic inference device has seen great advances in recent years (Chater & Oaksford, 2008; Friston & Stephan, 2007), allowing important aspects of the minimal and narrative self during perception and action to be considered in the light of how probabilistic prediction interacts with sensory evidence. The computations that brains perform to predict and hypothesis-test underlie what it is like to be an *I who expects* the consequences of acting and perceiving – now and through time (Hohwy, 2007). In this article, we extend this work to self-perception in the interpersonal domain, while acknowledging that the sense of self is important, even in the absence of interactions with others. Simple observation suggests that the interpersonal self is as complicated in its detailed mechanics as it is blatant about its presence. While the minimal self at the core of near-instantaneous perception is difficult to put into words, there is, in the first instance, nothing difficult about putting the interpersonal self into words: ‘am a kind person’, ‘am not as good as her’. The English language describes this powerful self-perception with expressions such as ‘he is a terribly self-conscious’.

We claim that the interpersonal self is *actively inferred* during social exchanges and that many of its properties *correspond to the means and ends of a machinery of probabilistic inference*. Inferring self-representations may thus *help achieve desirable ends* (social outcomes). The evidence that we marshal to develop this argument comes from a wide variety of sources, including computational neuroscience, brain imaging, psychiatry, social and clinical psychology. We develop our claim as follows. First, we provide evidence that some high-level, affectively coloured (pain) perception is well described in terms of basic Bayesian reasoning. Second, we describe an extended framework of approximate Bayesian reasoning, namely active inference, which encompasses agency and decision-making. Third, we review the kinds of psychological construct upon which active inference may operate – and show that inferring about these constructs subserves important goals. Fourth, we suggest a model of interpersonal exchange that could form the basis for empirical study. Finally, we examine the psychiatric relevance of making affectively charged inferences, especially about the self. We conclude by discussing the limitations of our approach and the implications for future research.

## 2 Simple Bayesian inference in high-level perception

### 2.1 Using Bayesian inference to make sense of experience

The Bayesian approach considers probabilities to be degrees of belief, so that Bayesian inference has the following form. If I make an observation *o*, what should become of my belief *P* (*S* = *s*) that some relevant aspect of the world is in state *s*? For example,^1^ if *o* = ‘Emil gave me a present’, what should become of my belief ‘I am a bad person’? If the new observation is surprising – with respect to the existing belief framework – the framework is poor at predicting the observation. It therefore needs to be updated if it is to describe the world more adequately. This updating of beliefs is the essence of Bayesian inference, which adjusts the agent’s model of the world so as to render new observations (data) less unpredictable. Although a full description of this well-established formal approach is outside the scope of the present article, the interested reader is referred to (Chater & Oaksford, 2008; Friston & Stephan, 2007; Friston et al., 2013; King-Casas et al., 2008). The claim we make in this paper is that this inferential framework applies to all beliefs – including beliefs about the self.

In a Bayesian framework *what the brain minimises as it makes inferences, including inferences about the self, is unpredictability* and not, for example, proximal discomfort. We will consider an example of this below, in the case of perception of pain. We reformulate the principle of psychological economy as follows: the *primary gain* of a representation is its power to predict outcomes that matter under some prior beliefs. Maximising predictability is equivalent to minimising surprise. Clearly, surprising outcomes rest upon prior beliefs. In our case, these beliefs will be about the self (and others). Crucially, surprise can be quantified as the negative log (Bayesian) evidence for a model. This means that minimising surprise maximises the evidence for a model or representation of interpersonal exchange.

We now turn to a simple but informative application of the Bayesian framework, the understanding of placebo responding. Placebo responding crucially depends on an interaction between prior beliefs about analgesia and sensory evidence (Morton, El-Deredy, Watson, & Jones, 2010). This case study will help structure further discussion in two ways: on the one hand, its limitations will motivate the need for goal-directed, *active* inference; but on the other, placebo-responding provides important lessons for inference about self-representations.

### 2.2 The Bayesian model of pain perception

The Bayesian model of pain perception^2^ (El-Deredy, Trujillo- Barreto, Watson, & Jones, 2010; Watson, El-Deredy, Bentley, Vogt, & Jones, 2006) provides proof-of-principle that humans perform high-level Bayesian inference to form affectively charged percepts. These researchers modelled pain perception in two groups of healthy people, ‘placebo responders’ and ‘placebo non-responders’. Pain ratings were collected through three phases. In both groups:

• Painful (‘active’) stimuli were initially administered in the absence of any treatment, establishing an expectation of stimulus – pain perception.
• Placebo analgesia was then administered while stimulation was covertly switched to sham stimuli. These were visually identical but non-painful, giving the impression that the ‘treatment worked’.
• Stimulation was finally covertly switched back to active stimuli.

Subjective pain ratings were collected throughout the experiment. A family of probabilistic models of pain perception were fitted to the data. The model for pain rating *p* of participant *s* at time *t* was:

(1) $$\begin{array}{cc} & p_{s\text{,}t}=c_{u\text{,}t}o_{t}+c_{y\text{,}t}p_{s\text{,}t-1}+\varepsilon \\ & \varepsilon ∼N(0\text{,}\beta_{t})\\ & c_{u\text{,}t}∼N({\overset{ˆ}{c}}_{u\text{,}t}\text{,}\alpha_{u\text{,}t})\\ & c_{y\text{,}t}∼N({\overset{ˆ}{c}}_{y\text{,}t}\text{,}\alpha_{y\text{,}t}) \end{array}$$

where ${\overset{ˆ}{c}}_{u\text{,}t}$ and ${\overset{ˆ}{c}}_{y\text{,}t}$ parameterise the weight that each participant attaches to their immediately preceding experience of pain *p_s_*_,_*_t_*_−1_ and to their sensation (observation) *o_t_* respectively. *β* is a precision (noise) parameter common to all participants while *α_u_*_,_*_t_*, *α_y_*_,_*_t_* are the precisions of the weights ${\overset{ˆ}{c}}_{u\text{,}t}\text{,}{\overset{ˆ}{c}}_{y\text{,}t}$. Precision is the inverse variance and encodes the expected reliability of a variable. In Bayesian inference, evidence that has a greater precision has more influence on beliefs. El-Deredy et al. used variational Bayes (variational free energy minimisation) to update $\beta_{t}$, the values of the weights ${\overset{ˆ}{c}}_{u\text{,}t}\text{,}{\overset{ˆ}{c}}_{y\text{,}t}$ and their precisions *α_u_*_,_*_t_*, *α_y_*_,_*_t_* on each trial.

The above Bayesian updating model of pain rating allows one to address the following question: given beliefs subjects already entertained (priors) about how painful percepts are generated, how are current percepts integrated into new beliefs (posteriors)? Crucially, ‘new beliefs’ include the *precision or the weight that should be attached to past reports.* The role of precision is crucial, because agents come to rely more on cues that have the greatest precision. Notice that the previous response enters as a new observation in this Bayesian updating scheme – in other words, the model is observing and trying to explain its own responses.

The authors found that the full model explained the pain-perception data of placebo non-responders, but a reduced model – *which neglects sensory information* (*o_t_*) *and makes predictions based only on past reports* – best accounted for the data from placebo-responders.^3^ In other words, one’s inference about the causes of nociceptive input can be based purely upon previous reports or, equivalently, behaviour that belies one’s own inferences. Bayesian updating thus provides a good account of behaviours involving high-level beliefs about analgesic properties. This example also provides proof-of-principle that apparently irrational, affectively charged human beliefs can be described quantitatively in terms of the balance of prior beliefs relative to sensory evidence.

Interpersonal interactions also involve apparently irrational, affectively charged beliefs and the related negative affect is neurally and subjectively related to pain (Eisenberger, 2012). Neither physical pain nor social ‘pain’ stand in a one-to-one relation with damage: people have widely varying sensitivities to the same physical manipulation (Eisenberger, Jarcho, Lieberman, & Naliboff, 2006). The experience of physical pain is not unrelated to self-awareness (‘Pinch me, am I dreaming?’) but interpersonal ‘pain’ is quite directly related to self- and other- representation. When I am deceived, for example, beliefs like ‘*I am* a fool’ and ‘*he is* devious’ immediately gain weight. Much as inferences about pain relate to the risk posed by stimuli (and, more subtly, my own physical vulnerability), so during interpersonal inference the self and others are vividly experienced as vulnerable (or not), noxious (or safe), etc. We will consider examples of inferring aspects of the self to be noxious, when we consider psychiatric conditions later on.

This brings us to a key limitation of this Bayesian model of pain perception, which is its silence as to the *functional role* of inference about pain (or, in our case, about the self). In fact the optimal readiness with which pain is to be inferred depends on context: organisms can tune their own pain perception according to both their prior beliefs and the specific biological goals they believe are attainable in that context (Boureau, 2005). This would be an irrational anomaly if pain were a raw datum, but not if pain was a motivational force: if there is little of biological use that can be done, it is better to reduce pain sensitivity. We therefore need a Bayesian framework that explicitly represents the person’s *agency* and *goals,* namely active inference (Friston et al., 2013). In order to study self-awareness as inference, we need to quantify the relevant aspects of self-representation. We will consider this in more detail below; for now, suffice it to say then we can cast traits like ‘fairness’ or ‘jealousy’ as social preferences: how much another’s pain or gain can act as a motivating force (Camerer, 2003). We propose that such sensitivities (preferences) may stand to social outcomes as pain sensitivity stands to somatic ones. That is, their usefulness may lie in helping the agent reach their goals.

## 3 Active Bayesian inference and self-representation

### 3.1 Using active inference to reach desirable goals

During perceptual inference, we infer those descriptions of the world that are most consistent both with our sensory data and with our prior beliefs about what the world should be like. During *active* inference, however, we can entertain beliefs about alternative scenarios *that lie ahead* – scenarios that depend on our own fictive choices in the future. Policies are then chosen in a Bayes optimal fashion that depends upon these prior beliefs. Our ‘prior beliefs’ are not just about what we believe the world to be like, but which alternative outcomes we realistically expect (hope) that we can reach: our *goals*. From now on we will refer to prior beliefs about goals simply as ‘goals’.

Conversely, once a subject has experienced part of the scenario, but has not as yet reached the final outcome, they can be said to hold *empirical priors* about their policy (i.e. the choices that are shaped by their goals). These empirical priors pertain to (a) goal attainability from the current state and (b) beliefs about future choices (Friston & Stephan, 2007; Friston et al., 2013). Empirical priors are a necessary aspect of inference in hierarchical models. Technically speaking, they are prior beliefs that are conditioned on (i.e. depend on) other unknown variables. In our case, beliefs about the policy depend upon beliefs about hidden states of the world, where beliefs about future states depend upon the goal. Whether I have to get off the bus may not just depend on whether I see a park from the bus window, it also depends on which bus I took! In this sense, beliefs about the policy entail beliefs about hidden states in a hierarchical sense and are therefore empirical priors. They are essentially prior beliefs about prior beliefs.

To make the distinction between goals and empirical priors over policies clearer, consider going to the pub for a drink. My favourite drink is actually fairtrade hot chocolate; I also like Guinness, but less so. If they were in front of me I would choose fairtrade chocolate ¾ of the time, Guinness ¼ of the time, so these are my goals: [0.75, 0.25]. This illustrates how – in our framework – all utilities are necessarily relative: the utility of a particular outcome is always defined in relation to allowable alternatives. The computational purpose of defining goal priors can be seen as separating out the various task-dependent probabilities used in active inference from probabilities that do not depend on where we are in the task or on the agent’s behaviour during the task. In other words, we separate state-dependent beliefs about what we will do next from the goals that define beliefs about the final outcome.

In contrast to goals, empirical priors entail beliefs about the dynamics of the task or, more simply, the consequences of a particular action in terms of the transition from one state to another: where will I end up if I take this sequence of actions? And how does the disparity between that outcome and my goals influence my choice of a subsequent policy? If I take a bus towards Barnet (action), am I likely to get to a nice pub (state) *and* look for hot chocolate *there* (policy)? In short, empirical priors entail the agent’s knowledge about the dynamics of her/himself in the world. They are ‘priors’ in the sense that the actual policy that we will choose (shall I look for hot chocolate?) will depend on observations about where we are and where we can go. Crucially, in the active inference framework, these choices will also depend on the confidence we have in reaching our goals from the current state. This is because action depends upon beliefs over policies and beliefs always have a precision.

Our inference framework is thus able to decide about actions motivated by goals that embody the raw power of gain and loss, contentment and pain. The key device to achieve this, introduced in this review, is to convert the utilitarian formulation of classical economic theory, in which choices are assumed to maximise expected utility, into a pure (Bayesian) inference problem. One can do this by representing the utility of outcomes as prior beliefs about final states. This means that instead of making choices to maximise expected utility, one simply minimises surprise – under the prior belief that one will end up in desirable states. Practically, replacing utilities with prior beliefs means that one can appeal to well-established inference schemes such as (variational) free-energy minimisation in order to prescribe normative behaviour.

Casting utility functions as prior beliefs means that one can understand utility – which depends on interpersonal factors – in terms of beliefs about oneself and others. As an example, assigning high utility to gains acquired by a partner who resembles me translates into holding a higher prior belief that the partner will be of a similar type to me *and* that they will gain from the exchange. So far we have only used common-sense examples; in order to apply the formalism of active inference to self- and other- representation, we must determine – at least roughly – the categories of belief about self and others that people actually use.

### 3.2 The nature of beliefs about the self (and others)

During self- and other-perception, empirical evidence suggests that people form beliefs about both the states and traits, of both self and others (Bem, 1972; Bentall, 2003; Fowler et al., 2006). Traits are characteristic patterns of emotion, behaviour or thinking that subjects engage across many situations. People naturally infer such traits in each other, in order to anticipate intentional mental states. Psychological research suggests that people independently score ‘positive attributes’ and ‘negative attributes’ of both ‘self’ and ‘other’. A modern clinical research instrument, used to assess such representations, is shown in Table 1. The items relate to (i) (social) outcomes; e.g. ‘respected’, ‘a failure’; (ii) Capabilities; e.g. ‘weak’, ‘talented’ and (iii) Preferences for acting according to social values; e.g. ‘good’, ‘fair’, ‘trustworthy’. Note that the instrument is asymmetric: ‘other’ attributes relate more to social-value preferences, such as fairness, harshness, etc.; whereas ‘self’ attributes relate more to social outcomes and abilities. This division is likely to be an artefact of the clinical focus of this scale; namely, depression and paranoia. If people are asked to describe ‘what kind of person’ they desire to be and ‘what kind of person they try to avoid being’, they give a mixture of success-, motivation- and ability-related traits for themselves too (Francis, Boldero, & Sambell, 2006). We will call a ‘type’ the traits of a person that are relevant to the current context (e.g. an interpersonal task).

Building on the work on inferring an *other’s* type (Ray, King-Casas, Montague, & Dayan, 2008), we hypothesize that people harvest observations to update their beliefs about their own type for good reason: *Self (and other) representations are tools facilitating the efficient computation of interpersonal behaviour.* An important psychological theory that makes contact with these issues is the ‘sociometer theory of self-esteem’ (Leary, Tambor, Terdal, & Downs, 1995). According to the sociometer account, self-esteem has an important functional role, which is to indicate one’s likely evaluation by the social milieu. The crucial corollary is that actions that improve self-esteem are rewarding because – if all goes well – they subserve socially sanctioned goals.

We generalise the ‘sociometer theory of self-esteem’ is to a ‘sociometer theory of self-representation’. Type-based interpersonal representations, of which (trait) self-esteem is only a subset, serve to optimise context-dependent social computations.^4^ Aspects of self-representation important for prediction may be: (i) how successful I have been in a given context so far; (ii) an appraisal of my capabilities; and (iii) what my (interpersonal) preferences are. Constructs such as ‘a bad person’ or ‘fair’, can inform optimal decision-making in a formal and fundamental fashion, despite the emotive and informal nature of these concepts. Our contention that (interpersonal) preferences should be the object of inference parallels Hohwy’s hypothesis that desires are inferred in the process of applying generative models of ourselves (Hohwy, 2007). The coherence of these models would be important for the construction of the ‘narrative self’.

In an interpersonal context, traits such as ‘talented’, ‘harsh’ etc. can feed directly into the outcomes that a person wants to reach (or indeed to avoid). In terms of active inference, agents can *inform goals* by the desirable self-representations that they are likely to reach as outcomes. An example is: “If the outcome of this exchange is that I cooperate *and* my partner runs off with the money, this would be evidence that *I am a fool*. This is highly undesirable – I’ll attach very low probabilities to outcomes implying that I’m a fool”.

We hypothesise that different people are equipped with different prior beliefs, acquired during their upbringing and built on a base of genetic preparedness. This allows modelling of individual variation, including variation in psychopathology. It provides a simple and graceful way to account for different types (phenotypes) in a quantitative and formal sense. Clearly, people may have a vast lexicon of potential traits to consider. We expect the ensuing paradigm could the used to inform those traits that are inferred, especially in clinical applications.

### 3.3 Self-representation and desirable goals of social interaction

Type-based representations provide a simple and formal belief space that people may use in categorising themselves and others – a belief space that can be tested empirically. These representations can be linked to the returns (e.g. utilities or prior beliefs) that people expect themselves and others to derive from social actions. An agent’s task is then to infer, or estimate, ‘types’ linked to utilities. If, for example, I infer that my partner is a ‘fair person’, this summarises an expectation: that they will avoid actions leading to inequitable returns for all involved – finding such actions aversive. If I estimate myself to be ‘highly competitive’, I expect to derive high utility from actions that lead to me doing better than others, even if my material returns are somewhat lower than under some other outcome (e.g., where I come second).

But why bother using person-representations as heuristics? Why not just calculate out how everyone should behave based on their self-interest? The tractability of optimal Bayesian inference is a key issue here; real agents must perform the requisite computations, which can be very hard. Type-based interpersonal representations can be likened to heuristics that facilitate *approximate* Bayesian inference. Here type-based representations can be a shortcut to tracing out a complex tree of possible future states and outcomes. In the ‘stag hunt’ game, for example, agents with limited depth-of-thought but equipped with ‘prosocial’ preferences make decisions equivalent to having greater depth-of-thought but no social biases (Yoshida, Dolan, & Friston, 2008). In such cases, explicit optimal solutions are often prohibitively complicated to compute. When framed in terms of heuristics or prior beliefs, approximate Bayesian inference provides a tractable and – in some instances – neuronally plausible scheme for optimisation.

This approach marks a significant departure from the traditional (normative) behavioural-economic approach, where an agent’s beliefs about their own type do not depend on their actions and *vice versa*. It also differs from traditional psychological approaches, where self-representations – including theories based on self-esteem or on unconscious representations – are seen as the object of a more-or-less self-contained homeostasis, rather than an explicit heuristic of how to behave optimally in social exchanges.

### 3.4 Self- and other-representation in a model Trust Task

Game-theoretical paradigms can induce people to infer traits in others and express their own traits in beliefs and behaviour. The ‘Trust task’ is a prototypical paradigm (King-Casas et al., 2008; Ray et al., 2008). In each of several rounds one of the participants, in the role of the Investor, is given a certain ‘wage’. They can invest any fraction *f_I_* of that ‘wage’ with the Trustee. The Trustee makes a profit, which is usually taken to be 200%. They then keep as much of this as they want and return to the Investor any fraction *f_T_* of the ‘wage’. How much will the Investor entrust to the Trustee? Repeated rounds allow each player to try to deduce the ‘type’ of their partners. Are they disposed to cooperate (*f_T_* > *f_I_*), or maybe ‘grab the money and run’ (*f_T_* ≪ *f_I_*)? Behavioural studies demonstrate healthy people often ‘repair cooperation’ that falters, but patients with borderline personality drift towards the Nash equilibrium, suppressing overall incomes (King-Casas et al., 2008). The Nash equilibrium has a paranoid flavour: in this game, it says that in the last round the Trustee will ‘take the money and run’, and therefore should not be trusted. The same logic holds for the penultimate round, and so on through a process of iterative backward inference to the very first one. Therefore the Investor should never trust any money to the Trustee.

The Trust Task has been extensively studied, and is attended by a large amount of psychological and neurobiological data (Xiang, Ray, Lohrenz, Dayan, & Montague, 2012). However, existing models do not make adequate contact with clinical psychology. Here, we consider a framework for modelling this task that is equipped with minimal clinically relevant self- and other-representations. This can be used to analyse the behaviour of people who might hold unstable or distorted interpersonal representations. Our framework extends classical models (Camerer, 2003; Ray et al., 2008) that lack flexible, computationally functional self-representations.

Let us consider agents who think about alternative future scenarios to a limited depth-of-thought (‘one, two steps …’) and then consider how they and their partners would ‘come across’ in the long term (‘… infinity’). This gives interpersonal representations a functional role. Unlike classic neuroeconomic models – and closer to clinical psychology – at the end of each round subjects have to update their *own* self-image based upon their actions and those of their partner. We therefore have a Bayesian *Attribution – Self-Representation* (BASR) model, formalising the attribution theory suggested by Bentall (2003).

**Fig. 1:**
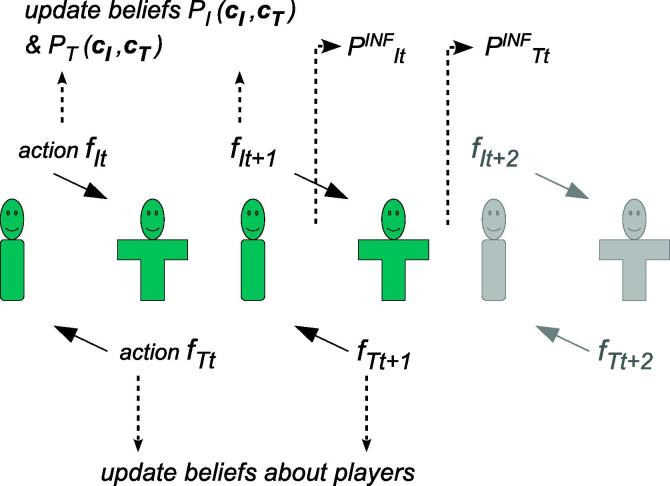
In an attribution-representation model, each partner considers the ‘character traits’ of both themselves and the other (***C**_I_*, ***C**_T_*), and forms corresponding beliefs ***P**_I_* (***C**_I_*, ***C**_T_*), ***P**_T_* (***C**_I_*, ***C**_T_*) about them. They infer the likely next move of both players and in the light of this they choose the actions to take at time *t*, ***f_It_***, ***f_Tt_***. After each character has taken a turn, both players update their beliefs – but, of course, each with their own priors. Partners are only able to calculate a small number of moves into the future. Most importantly, at time *t* they compare their ‘long term’ beliefs about outcomes, $P_{I}^{\mathrm{INF}}$, $P_{T}^{\mathrm{INF}}$ with their desired (prior) probability distributions over outcomes. The cycle repeats at *t* + 1 albeit in a curtailed form, without making inferences about behaviour at the next step, if this is the last round of the exchange.

In the BASR model, working out each possible scenario two moves into the future enables the ‘type’ of each partner at that future point to be estimated (Fig. 1). There are at least two interesting ways in which the estimated types of self and other may be useful. First, people may have intrinsic preferences as to the type of person they want to be, and the type of person they want their partner to be. The framework of active inference allows us to think about ‘wanting the partner to be’ as ‘believing that the partner will indeed turn out to be’. The preferences inform *goals* over the sort of person they are. We could, furthermore, hypothesize that such goal preferences are context specific: contexts may, for example, be labelled as competitive or cooperative, and stronger priors attached to corresponding person types. Second, types can be exploited in explicit approximations of the consequences of behaviour. An expectation that long-term behaviour will be consistent, on average, with the inferred player ‘types’ would lead to an estimate of long term returns. This would then be absorbed into appropriate goal priors.

Overall, the BASR model of a simplified Trust Task would run as follows. First, agents know what actions are available. Let’s allow them just two options in each round of 30 rounds, a high (cooperative) or low (uncooperative) contribution *f_high_* or *f_low_*. Second, agents hold beliefs about how ‘cooperative’ each player is, i.e. the player’s types. We can think of ‘cooperative’ being a positive, high-esteem trait in this context. Third, agents estimate the likely evolution of the game a small number of moves into the future – e.g. ‘I play X, she plays Y, then I play Z’. This evolution may be more or less likely according to the agents’ empirical priors. It may also be more or less compatible with their goal priors, as we saw. Agents also have a sense of how precise their prediction of reaching their goals may be; this ‘precision’ is important for the mechanics of active inference but outside the scope of this review. The interested reader is referred to our technical exposition (Friston et al., 2013). Finally, the player whose turn it is to play infers an updated view of the interaction. This maximises the consistency between their prior beliefs about the world, their observations, their goals, their confidence (precision) in reaching those goals – and of course their policy. Their action then flows from their preferred policy, and it’s the other player’s turn.

## 4 Clinical importance

### 4.1 The interpersonal self in psychiatric disorders

Severe psychiatric disorders often illustrate terrible distortions and deficits in self-perception. Let us consider two examples:

*“I cannot live and I cannot die, because I have failed so much, I shall bring my husband and children to hell ... I shall go to the convict prison and my two girls as well, if they do not make away with themselves because they were born in my body.”*

This extract is from a letter written by a severely depressed patient of Emil Kraepelin – the patient who failed to update her self-image in response to being given a nice present. It expresses a nihilism that remains familiar to contemporary clinicians, who are all too aware that a distorted representation of the self can be a debilitating symptom in severe depression. In the second example, self-perception is deficient:

*“Isn’t America supposed to be the land of the free? How come, if I’m free, I can’t deprive a stupid f^∗∗∗^ing dumbs^∗∗^t of his possessions if he leaves them on the front seat of this f^∗∗∗^ing van out in plain sight...? Natural selection. F^∗∗∗^er should be shot.”*

This is a quote from Mr. E. Harris, a mass murderer who attracted a diagnosis of psychopathic disorder (Cullen, 2009). It illustrates how he both talked and acted as if his representations of other people carried no value. This was most striking when the prospect of their suffering seemed to hold no aversive value for him. Moreover, he was devoid of any concern that his beliefs and behaviour would devalue him as a person, de facto rendering him despicable in any *community*.

Contemporary psychiatric practise acknowledges the importance of self-representation. Descriptive diagnostic criteria, such as the DSM-5, define a disturbance of the representation of self and the evaluation of others as core features of Personality Disorders (Skodol et al., 2011). Moreover, several empirically validated therapies assume that self-representations are important links in a causal chain that leads to psychiatric disorders. For example, cognitive-behavioural therapy postulates that ‘core beliefs’ related to the self, such as “I am unworthy”, underpin depression (Waller, Shah, Ohanian, & Elliott, 2001). Many mentalization-based therapies propose that infants internalise representations that caregivers make available to them – a process which, if it goes awry, may lead to conditions such as borderline personality disorder (Allen, Fonagy, & Bateman, 2008). The less evidence-laden approach adopted by psychoanalysis posits fragmented representations of others (part-objects) as underlying severe mental disorders. In Borderline Personality disorder, patients often fail to predict the damage that their actions cause, in terms of the way they are perceived by other people and the ruinous consequences this has for the person themselves (Allen et al., 2008).

### 4.2 When self and others are perceived as noxious entities

Many psychiatric conditions might be described as ‘nocebo states’. In nocebo states physiologically inert stimuli have aversive effects, e.g. an inert tablet causing nausea. This is a mirror image of the placebo effect. In psychiatry, features of the self or the environment that most outsiders would regard as innocuous are often perceived as toxic. A common example of perceiving a facet of the self as toxic is as follows. A patient with borderline personality quarrels with a relative about an everyday matter. This activates loathing of the self to such a degree that the patient hides away and carves insults on her skin with a razor. However most common, distressing ‘nocebo’ perceptions in psychiatry are unwarranted (to the outside observer) versions of everyday concerns – such as that others are devious and the self is worthless, as exemplified by the study by quoted in Table 1 (Fowler et al., 2006).

In many clinical accounts it is not only self-representations that are dysfunctional: efforts to bolster specific aspects of self-representation are also seen as maladaptive. However, the precise role of self-representation is highly controversial, partly due to the fact that it is difficult to quantify and access in a strictly empirical manner. This highlights the fact that despite a focus on self-representation over recent years, its normative function is poorly understood. There is obvious value for interpersonal exchange in asking “what sort of person is the *Other*?” However, the value of healthy inference about “what sort of person do these actions make *Me*” has not being addressed so thoroughly.

The subtle clinical concept of ‘mind-blindness’ is relevant here. Clinicians mean by this that borderline patients, especially under stress, show an inability to reflect about other people’s minds or indeed their own. Increasing this ability through gradual learning is the basis of Mentalization Based Therapy (Allen et al., 2008). As we saw, placebo responders are characterised by the absence of a term reporting sensory information about the outside world (no term in *y_s,t_*_−1_ in Eq. (1)). There are indications that people with borderline personality disorder may have an analogous but more subtle deficit in interpersonal interaction. When interpersonal Trust falters they fail to ‘signal’ to their opponent, by risking part of their income, that they are trustworthy. This leads to an unravelling of trust. In addition, healthy people playing borderline partners display a reduced level of theory-of-mind, as would be expected if the borderline partner were deficient in higher-order theory-of-mind terms (Xiang et al., 2012). The ‘missing term’ in these patients’ model of the world would be exactly “what sort of person would these actions make *Me?*” We thus have a computational neuroscience counterpart of the clinical concept of ‘mind-blindness’.

Estimation of social threat and opportunity using active inference requires not only prior beliefs about self and others, but also their approximate Bayesian updating – including the evaluation of alternative future scenarios. Several cognitive deficits and biases, such as mildly reduced IQ and working memory or a ‘jumping to conclusions’ cognitive style, are associated with paranoid psychosis (Bentall et al., 2009). How such non-specific cognitive deficits may contribute to the aetiology of abnormal beliefs is the subject of much debate. Affective biases are likely to involve prior beliefs about the nature and likelihood of noxious states in self and others. Could *nonspecific cognitive deficits shift interactions from cooperative to suspicious in the presence of a specific distributions of prior beliefs*? The cognitive-deficit part of this would be analogous to the way that play can shift from cooperative to non-cooperative under the influence of reduced depth-of-thought (Yoshida et al., 2008). Paranoia may result from prior beliefs about self and others that are activated by specific, aversive contexts – and maintained by a reduced cognitive ability to consider more complicated but benign scenarios. An important factor in generative models of paranoia may be the belief that persecutors do not attach aversive motivational value to the persecuted person’s distress. Such a model is actually true if one’s partner has psychopathy. The hallmark of this is that the partner is unable to attach aversive motivational value to the suffering of others (like Mr. Harris above) despite being able to infer its presence.

## 5 Challenges and limitations

### 5.1 The embarrassment of Bayesian riches

Active inference assumes that subjects optimise a probabilistic representation of the environment. This assumption is not one of perfect or rational behaviour but of a type of bounded rationality based upon a potential universe of prior beliefs or heuristics. Previous research has also used this philosophy, of perfect optimisation within a suboptimal cognitive model (Moutoussis, Bentall, El-Deredy, & Dayan, 2011). When it comes to psychopathology, however, we encounter a difficult problem: Is ‘deviant’ behaviour the result of inefficient model optimisation or due to an ‘inefficient’ model of the world? In other words, are prior beliefs (the model) abnormal, or is psychopathology the result of ‘broken’ active inference? This is a particularly important and challenging question as on the one hand, *any* behaviour can be explained as optimal Bayesian inference given *some* set of priors and utility function; while *some* behaviours are undoubtedly the result of what we may call ‘broken’ active inference. Examples of the latter are likely to include drug-induced psychosis or dementia.

### 5.2 How can one’s type be unknown?

If, as we maintain, a person’s type enters into their decisions, isn’t that person’s type tautologically the one which determined those decisions? In what sense can such a type be unknown to the subject, and have to be inferred?

At least three alternatives should or could be tested. First, there may be separate decision-making and self-evaluating parts of the self, something akin to an actor-critic architecture in reinforcement learning (Sutton & Barto, 1998). Parenthetically, no self-*deception* needs to be invoked here. Beliefs posterior to observing the world, including the self, need not accord with the maximum likelihood of observations: other constrains enter inference too, as we have seen in some detail. Second – in a related vein – there may be no well-defined ‘type’. The history of psychology and psychiatry is littered with abandoned models of personality traits and types, while stereotypes about groups have little predictive value. Like these models, people’s self-representations (e.g. ‘I am a music-lover’) may simply be instances of the fundamental attribution error. In this case, the approach suggested above might help delineate stable patterns of interactions and attributions, including self-fulfilling prophesies, which describe the situation at hand but have no deeper explanatory power. Third – and most interesting – types may have to be inferred and applied *in relation to social norms*. How much work a person has to do for others to be considered ‘kind’, or what offers a ‘fair’ person might make in an ultimatum game? (Xiang, Lohrenz, & Montague, 2013). If ‘kind’ or ‘fair’ people are treated on average better than ‘unkind’ or ‘unfair’ ones, then inferring one’s own type becomes even more important, and more challenging, for each social partner.

## 6 Conclusions: A program for characterising interpersonal representations

In conclusion, we have reviewed two key hypotheses. First, that people make inferences about themselves – and others – to minimise interpersonal surprise enabling them to make decisions that are most consistent with their model of the interpersonal world. Second, we suggest that the framework of active inference can capture the iterative dynamic of ‘where do *I desire* to get’, ‘what do *I decide*’ and ‘what do *my actions make me*’.

The programme of research that this analysis speaks to is to characterise different prior beliefs (about contingencies, utilities, prosocial utilities and types) in relation to choice behaviour, under the assumption that subjects engage in approximate Bayesian inference. Bayesian subjects are assumed to update of models of their world, while Bayesian researchers infer models of individual variability of choice behaviour (we could call the latter meta-Bayesian fitting). Both can be implemented through free energy minimisation. The updating of self-representations by subjects could then be mapped onto neurobiological substrates, e.g. via functional neuroimaging. This approach could help advance our understanding of distorted self- and other-representations in many conditions – including depression, personality disorder and paranoia.

Practically, we envisage that the first step will entail characterising the updating of beliefs over other people’s type, which in turn results from exposure to others’ actions, under the simplification that only the immediate returns matter. One could then consider prior beliefs about outcomes in the long-term future, and their influence on self-representation. Finally, one might simulate and study the behaviour of interacting self-representing agents that can even exert choice over the types of ‘games’ they engage in. We hope to pursue these studies and the ideas reviewed above to help contextualise and motivate a formal focus on how we represent ourselves and how these representations determine our behaviour.

## Figures and Tables

**Table 1:** Empirically validated attributes that people use – relevant to depression and paranoia – from the ‘Brief Core Schema Scale’ (Fowler et al., 2006). Despite the strongly related content, self/other and positive/negative behave as separate factors.

Person	Valence			
	Positive		Negative	
I am …	Talented	Interesting	Weak	Vulnerable
	Respected	Valuable	Unloved	Worthless
	Good	Successful	Bad	A failure

Other people are …	Fair	Truthful	Devious	Hostile
	Supportive	Trustworthy	Nasty	Harsh
	Good	Accepting	Bad	Unforgiving

## Footnotes

1 This is a real example, as will be discussed in the section on clinical implications of our proposal.

2 A detailed report on this model is available from Prof. El-Deredy (Wael.El-deredy@manchester.ac.uk) or through the University of Manchester (http://www.escholar.manchester.ac.uk/jrul/search/).

3 This past-experience driven model diverges from the experimental data during the very first trial when painful stimulation is covertly switched back on.

4 For the sake of simplicity we do not discuss the time dependence of what we call traits and types here. We do not claim that self-esteem has a similar time-dependence as, say, extraversion.
